# Low Expression of miR-196b Enhances the Expression of *BCR-ABL1* and *HOXA9* Oncogenes in Chronic Myeloid Leukemogenesis

**DOI:** 10.1371/journal.pone.0068442

**Published:** 2013-07-19

**Authors:** Yue Liu, Wenling Zheng, Yanbin Song, Wenli Ma, Hong Yin

**Affiliations:** Institute of Genetic Engineering, Southern Medical University, Guangzhou, China; UCSF/VA Medical Center, United States of America

## Abstract

MicroRNAs (miRNAs) can function as tumor suppressors or oncogene promoters during tumor development. In this study, low levels of expression of miR-196b were detected in patients with chronic myeloid leukemia. Bisulfite genomic sequencing PCR and methylation-specific PCR were used to examine the methylation status of the CpG islands in the miR-196b promoter in K562 cells, patients with leukemia and healthy individuals. The CpG islands showed more methylation in patients with chronic myeloid leukemia compared with healthy individuals (P<0.05), which indicated that low expression of miR-196b may be associated with an increase in the methylation of CpG islands. The dual-luciferase reporter assay system demonstrated that BCR-ABL1 and HOXA9 are the target genes of miR-196b, which was consistent with predictions from bioinformatics software analyses. Further examination of cell function indicated that miR-196b acts to reduce BCR-ABL1 and HOXA9 protein levels, decrease cell proliferation rate and retard the cell cycle. A low level of expression of miR-196b can cause up-regulation of BCR-ABL1 and HOXA9 expression, which leads to the development of chronic myeloid leukemia. MiR-196b may represent an effective target for chronic myeloid leukemia therapy.

## Introduction

MicroRNAs (miRNAs) are non-coding single-stranded RNAs of 19 to 25 nucleotides. They are involved in a variety of biological processes and function by binding to target mRNAs to cause degradation or inhibition of translation. Studies have shown that miRNAs are involved in tumorigenesis and can act as tumor suppressors or oncogene promoters [1]. MiRNA molecules can be regulated by epigenetic processes such as methylation, which can also be involved in tumor initiation and development [2].

The miRNA, miR-196b, is closely associated with some types of leukemia. Overexpression of this miRNA significantly delays mixed lineage leukemia-fusion-mediated leukemogenesis in primary bone marrow transplant patients [3]. In addition, miR-196b has been shown to be down-regulated in EB-3 cells and in patients with B-cell acute lymphocytic leukemia (ALL). These data indicate that miR-196b could be a potential therapeutic target in B-cell ALL [4]. In contrast, miR-196b was over-expressed in patients with acute myeloid leukemia (AML) and the carcinogenic *NPM1* mutation [5].

Little is known of the role of miR-196b in chronic myeloid leukemia (CML). In this study, we have demonstrated that the expression of miR-196b is lower in CML patients than in healthy individuals. The *BCR-ABL1* gene and *HOXA9* gene have been identified as likely targets of miR-196b, using bioinformatics software [6]. Both *BCR-ABL1* [7] and *HOXA9* [8] have already been shown to be associated with CML. We therefore hypothesize that miR-196b acts as a tumor suppressor in CML, via the up-regulation of *BCR-ABL1* and *HOXA9*. We have also investigated the role of epigenetic regulation in the decreased expression of miR-196b in CML.

## Materials and Methods

### Detection of miR-196b expression in clinical samples

The bone marrow from 16 patients with CML and 10 healthy age-matched controls, which obtained signed informed consent and approval by the institutional review board (Nanfang Hospital Medical Ethics Committee), was used for the preparation of mononuclear cells, using Ficoll-Paque PLUS (GE Healthcare, Piscataway, NJ, USA). Total RNA was extracted using TRIzol (Invitrogen, Carlsbad, CA, USA), in accordance with the manufacturer's instructions. Reverse transcription of cDNA was performed from total RNA samples, using specific miRNA primers from a Hairpin-it miRNAs qPCR Quantitation Kit (Genepharma, Shanghai, China). Real-time quantitative polymerase chain reaction (RT-qPCR) was used to quantify miR-196b expression, with a specific stem-loop primer from the Hairpin-it kit and an Applied Biosystems 7500 system (Applied Biosystems, Carlsbad, CA, USA). The small nuclear RNA, Cel-mir-39, was used as an internal reference for relative miRNA quantification. The primer sequences are shown in Table S1 in File S1.

### CpG island detection

The CpG Island Searcher software (http://www.cpgislands.com/) [9] was used for the detection of CpG islands. The search parameters were as follows: GC content >55%; ratio of CpG observed versus CpG expected >0.65, length >500 base pairs.

### Epigenetic drug treatment of K562 cells

K562 cells were purchased from the American Type Culture Collection and treated in three groups; 1. Treated with demethylation drug 5-Aza-2′-deoxycytidine (Aza, Sigma, St. Louis, MO USA), 2. Treated with histone deacetylase inhibitor 4-Phenylbutyric acid (PBA, Sigma), 3. Treated with both Aza and PBA (Aza+PBA). The experiment was performed as described previously, using Cell Counting Kit-8(CCK-8, Dojindo, Kumamoto, Japan) [10], [11]. DNA was extracted 72 h after treatment, using the DNA and Blood Mini Kit (Qiagen, Valencia, CA, USA), as described by the manufacturer.

### Measurement of miR-196b promoter CpG island methylation status in K562 cells, by bisulfite genomic sequencing polymerase chain reaction (BSP)

Genomic DNA (1 μg) was treated with bisulfite, in accordance with the manufacturer's protocol (Qiagen EpiTect Bisulfite kit), and eluted in a total of 40 μl of elution buffer. The resulting DNA (2 μl) was used as the template in a 25-μl PCR reaction. Touchdown PCR was performed as follows: 98°C for 4 min; 20 cycles of 94°C for 45 s, 66–56°C for 45 s, with a 0.5°C reduction every cycle, and 72°C for 1 min; 20 cycles of 94°C for 45 s, 56°C for 45 s and 72°C for 1 min; a final extension at 72°C for 8 min. Bisulfite PCR products were gel purified (Promega, Fitchburg, WI, USA) and cloned into the pUC18-T plasmid (Sangon Biotech, Shanghai, China). Independent clones were sequenced using a BiQ analyzer (v2.00) [12]. Primer sequences are given in Table S1 in File S1.

### Measurement of miR-196b promoter CpG island methylation status in clinical samples, by methylation-specific polymerase chain reaction (MSP)

The DNA was extracted from the bone marrow of 45 leukemia patients and 10 healthy controls and then treated with bisulfite (EpitTect Bisulfite Kit, Qiagen). Promoter methylation of miR-196b was detected by PCR, using M- and U-PCR primers (Table S1 in File S1) to specifically recognize methylated or un-methylated versions of the promoter. The annealing temperature was 60°C for M-PCR, and 55°C for U-PCR, with 25 cycles used for each.

### Bioinformatics prediction of miRNA binding sites

TargetScan (release 5.2) [13], PicTar [14], miRanda (August 2010 release) [15] and miRNA Viewer (April 2005 version) [16] were used to predict miR-196b binding sites.

### Plasmid constructs and luciferase assay

Total RNA was isolated from K562 cells using TRIzol (Invitrogen). Reverse transcription-PCR was then used to amplify the 3′-UTR (untranslated region) of human *BCR-ABL1* mRNA (1,988 base pairs). The M-MLV RTase cDNA Synthesis kit (Invitrogen) was used for this reaction. Next, Subcloning was performed by using gene splicing, with an overlap extension from the *BCR-ABL1* mRNA 3′-UTR or the *HOXA9* mRNA 3′-UTR. The resulting PCR product contained miR-196b, which was combined with the loci seed sequence mutation in the *BCR-ABL1* 3′-UTR and *HOXA9* 3′-UTR. The subcloned fragments were inserted into the reporter psiCHECK™-2 construct (harboring Renilla and firefly luciferase genes) [17] by double digestion with Xho I and Not I (New England Biolabs, Ipswich, MA, USA). The assembled constructs were then sequenced. Primers are displayed in Table S1 in File S1.

Co-transfection of 0.8 μg of the plasmids and 50 nM solutions of miR-196b mimics (Genepharma), into 293T cells (purchased from the American Type Culture Collection), was achieved using Lipofectamine 2000 (Invitrogen), in accordance with the manufacturer's instructions. The control supplied with the RNA mimics and transfection with Lipofectamine 2000, alone (no plasmid or miR-196b), were used as assay controls. Forty-eight hours after transfection the luciferase assay was performed using the dual-luciferase reporter assay system kit (Promega) and Infinite M200 (Tecan, Männedorf, Switzerland). A decrease in luciferase activity indicated degradation of the target (*BCR-ABL1, HOXA9*) mRNA. The experiments were performed in triplicate.

### Plasmid constructs and lentivirus production

Healthy human bone marrow samples were used for DNA extraction. The human pre-miR-196b (284 base pairs) region was amplified and the PCR fragments were assembled with the plasmid pLVTHM, after double digestion with Mlu I and Cla I (New England Biolabs). The assembled constructs were then sequenced. The primers are shown in Table S1 in File S1.

The lentivirus vector was generated as described previously [18], with omission of the concentration step. The vector was classified as a 196b virus and a pLV virus. Cells that were infected with lentivirus were sorted by fluorescence-activated cell sorting (FACS). Green fluorescence indicated that cells were over-expressing miRNA-196b and pLV virus.

### Transfection

Cells that were infected with lentivirus were grown in RPMI-1640 culture medium (Gibco, Grand Island, NY, USA), with 10% fetal bovine serum (Gibco), at 37°C in a 5% CO2 atmosphere. Transfection with 100 nM solutions of miR-196b inhibitors (Genepharma) was performed using Lipofectamine 2000 (Invitrogen), in accordance with the manufacturer's instructions, using 4×10^5^ cells in six-well plates. The control supplied with the RNA inhibitor and transfection with Lipofectamine 2000, alone, served as controls for the assay.

The K562 cells were transfected with 50 nM solutions of *BCR-ABL1* siRNA (*ABL1*-homo-265, Genepharma) and 50 nM solutions of *HOXA9* siRNA (*HOXA9*-homo-452, Genepharma), using Lipofectamine 2000 (Invitrogen), in six-well plates (Table S2 in File S1). Control siRNA and transfection with Lipofectamine 2000, alone, were used as the controls for the assay. The cells were collected 48 h after transfection.

### Cell proliferation and cell cycle assays

The proliferation rates of K562 cells and cells infected with lentivirus were quantified in 96-well plates, using the CCK-8 kit (Dojindo), in accordance with the manufacturer's instructions. Measurements were taken every 24 h for a total of 5 days. The proliferation rates of transfected cells were examined after 48 h. Proliferation under different conditions was assessed in triplicate. Cell cycle assays were performed as described in a previous study [19].

### RT-qPCR analysis

Detection of miR-196b followed a similar method mentioned above, except that U6 was used as an internal reference. The primers are shown in Table S1 in File S1.

### Western blot analysis

Total protein was prepared and quantified as described in a previous study [20] and then transferred to a polyvinylidene difluoride membrane (Millipore, Bedford, MA, USA). The membranes were probed with antibodies for BCR-ABL1 (sc-23, 1∶500, Santa Cruz Biotechnology, CA, USA), HOXA9 (sc-81291, 1∶500 dilution, Santa Cruz Biotechnology) and β-actin (sc-130301, 1∶2,500 dilution, Santa Cruz Biotechnology, used as a gel loading control). An enhanced chemiluminescence fluorescence system (Thermo Scientific Pierce, Waltham, MA, USA) was used to detect antigen–antibody interactions.

### Patient samples

Bone marrow samples were collected from 16 patients with CML, 14 patients with AML, 15 patients with ALL and 10 healthy, age-matched, controls (Table S3 in File S1). Informed consent was obtained from all individuals and approval was obtained by the institutional review board (Nanfang Hospital Medical Ethics Committee). The diagnosis of leukemia was based on clinical features and hematological characteristics, in accordance with the National Comprehensive Cancer Network Guidelines.

### Statistical analysis

The results of MSP were analyzed by multi-sample rate χ2 tests, using SPSS 13.0 software. The results of the luciferase assay and cell proliferation detection assay were analyzed by one-way ANOVA, using SPSS 13.0 software.

## Results

### Methylation of CpG islands in the miR-196b promoter

**Figure 1:**
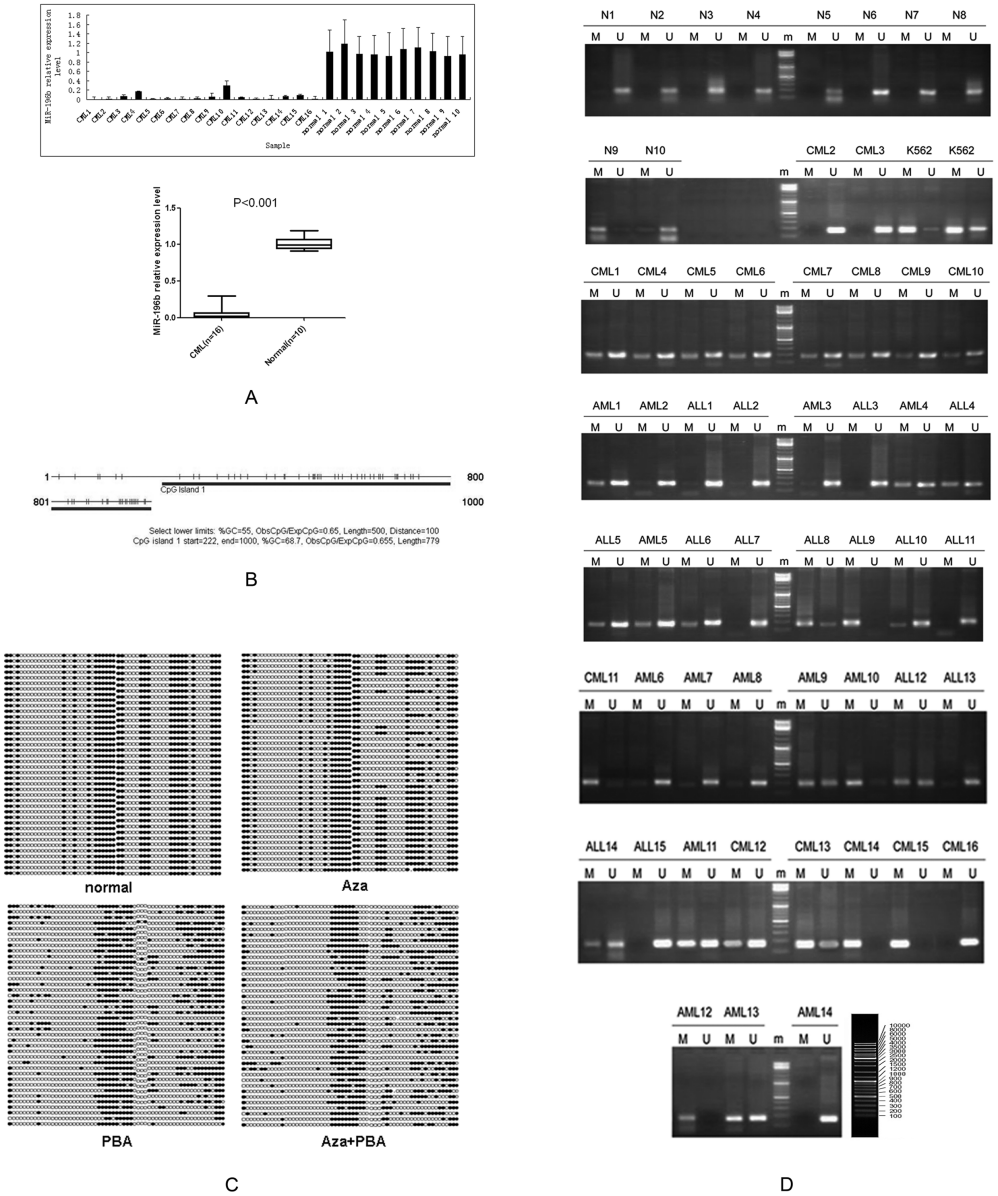
MiR-196b expression and CpG island methylation. (A) MiR-196b expression levels were significantly lower in CML patients than in healthy controls. (B) Predicted miR-196b promoter CpG islands, obtained from CpG Island Searcher software. (C) The BSP detection method demonstrated decreased methylation after treatment. (D) MSP test results for 45 patients with leukemia and 10 healthy controls. N represents the healthy controls.

The average expression level of miR-196b was significantly lower (P<0.001) in the bone marrow samples from the 16 CML patients compared with the 10 healthy controls, as indicated by the RT-qPCR (Figure 1A). In light of this, we investigated the role of epigenetic mechanisms that may be involved in the silencing of miR-196b. A CpG island, similar to that present in many tumor suppressor genes, was found in the 1000 bases upstream from the transcription start site of miR-196b (Figure 1B).

The miR-196b CpG island methylation status, in K562 cells treated with 15.55 nM Aza, 1.5 nM PBA, and 15.55 nM Aza+1.5 nM PBA, was examined using BSP. The results indicated that methylation was decreased after all the treatments, particularly after the combined Aza + PBA treatment (Figure 1C).

MSP was used to detect methylation of the 423–591 bases upstream from the transcriptional start site of miR-196b in 45 patients with leukemia and 10 healthy individuals (Figure 1D). Multi-sample rate comparison χ2 tests showed sample to sample differences in CpG island methylation status (P = 0.005; Table 1). A significantly higher level of methylation was observed in CML patients compared with the healthy controls (P = 0.001).

**Table 1:** Analysis of CpG island methylation status by MSP.

Typing	Methylation +	Methylation −	Total	?^2^	P(two sides)
CML	13(81.3%)	3(18.8%)	16	12.652	0.005
AML	8(57.1%)	6(42.9%)	14		
ALL	8(53.3%)	7(46.7%)	15		
healthy controls	1(10%)	9(90%)	10		

Six pairwise comparisons were performed, and the significance level was adjusted to 0.0083, in accordance with the Bonferroni correction. CML vs. healthy controls, Fisher's exact test, P = 0.001; AML vs. healthy controls, Fisher's exact test, P = 0.033; ALL vs. healthy controls, Fisher's exact test, P = 0.040; CML vs. AML, Fisher's exact test, P = 0.236; CML vs. ALL, Fisher's exact test, P = 0.135; AML vs. ALL, P = 0.837.

### Prediction of target genes

**Figure 2:**
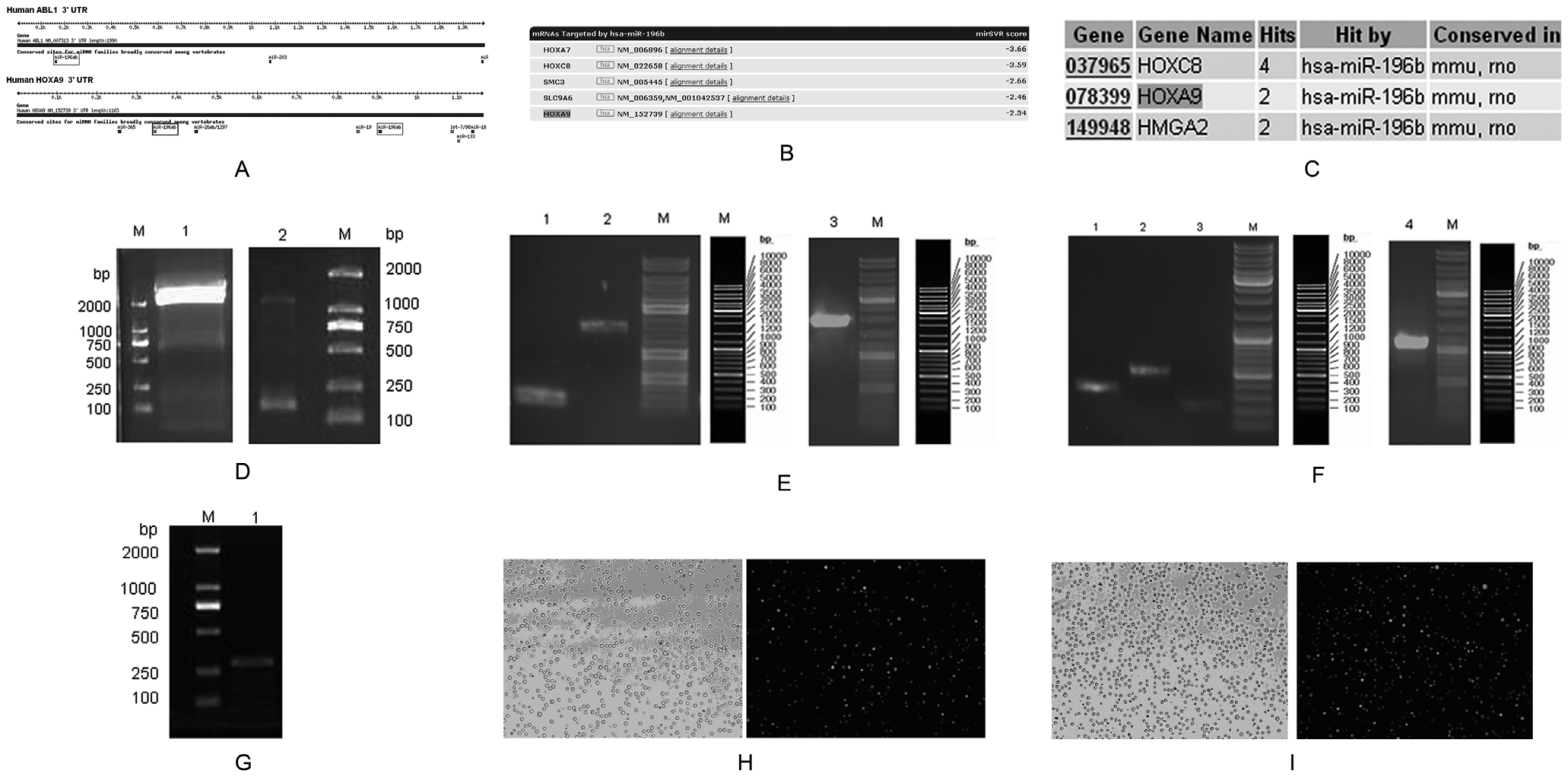
Target gene prediction, plasmid constructs and lentivirus infection. (A) Target genes of miR-196b, predicted by TargetScan. (B) Target genes of miR-196b, predicted by miRanda. (C) Target genes of miR-196b predicted by miRNA Viewer. (D1): *BCR-ABL1*-3′-UTR, (D2): *HOXA9*-3′-UTR. (E1): *BCR-ABL1*-3′-UTR-mut-1, (E2): *BCR-ABL1*-3′-UTR-mut-2, (E3): *BCR-ABL1*-3′-UTR-mutant. (F1): *HOXA9*-3′-UTR-mut-1, (F2): *HOXA9*-3′-UTR-mut-2, (F3): *HOXA9*-3′-UTR-mut-3, (F4): *HOXA9*-3′-UTR-mutant. (G): pre-miR-196b. (H): K562 cells infected by 196b virus (10×). (I): K562 cells infected by pLV virus (10×).

The TargetScan software predicted that *BCR-ABL1* and *HOXA9*, which are closely associated with CML, are target genes of miR-196b (Figure 2A). Both miRanda (Figure 2B) and miRNA Viewer (Figure 2C) software predicted that the target genes of miR-196b included *HOXA9*. In contrast, the PicTar software failed to identify any target genes of miR-196b. Overall, these results indicated that *BCR-ABL1* and *HOXA9* are likely to be target genes of miR-196b.

### Validation of miR-196b target genes by the dual-luciferase reporter assay

The 3′-UTR of human *BCR-ABL1* mRNA (Figure 2D1) and *HOXA9* mRNA (Figure 2D2) were amplified from the total RNA of K562 cells. The *BCR-ABL1* 3′-UTR-mutant (Figure 2E3), which contained one combined loci seed sequence, was subcloned by division into two segments (Figure 2E1, 2E2). The *HOXA9* 3′-UTR-mutant (Figure 2F4), which contained two combined loci seed sequences, was subcloned by division into three segments (Figures 2F1, 2FC2, 2F3). The data from the assay were analyzed using one-way ANOVA, which revealed unequal variances between treatment factors in the miR-196b mimics plus *BCR-ABL1* 3′-UTR group (P<0.001). The results of multiple comparisons showed that miR-196b mimics plus *BCR-ABL1* 3′-UTR significantly decreased the fluorescence value (P<0.05), which indicated the inclusion *BCR-ABL1* in the target genes of miR-196b. Unequal variances were also observed between treatment factors in the miR-196b mimics plus *HOXA9* 3′-UTR group (P<0.001). Again, the results of multiple comparisons showed that miR-196b mimics plus *HOXA9* 3′-UTR caused a decrease in the fluorescence value (P<0.05), which supports the hypothesis that *HOXA9* is a target gene of miR-196b (Table 2).

**Table 2:** Dual-luciferase reporter gene assay.

	n	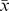 ±*s*
miR-196b mimics+*BCR-ABL1*-3′-UTR	3	532.793±111.245*
miR-196b mimicscontrol+*BCR-ABL1*-3′-UTR	3	1497.843±204.271
miR-196b mimics+*BCR-ABL1*-3′-UTR -mutant	3	1523.627±109.590
lipo2000+*BCR-ABL1*-3′-UTR	3	1488.233±94.823
lipo2000+*BCR-ABL1*-3′-UTR -mutant	3	1473.147±158.225
F	45.234	(welch)
P	<0.001	
miR-196b mimics+*HOXA9*-3′-UTR	3	507.629±32.345^#^
miR-196b mimics contro+*HOXA9*-3′-UTR	3	1160.171±60.471
miR-196b mimics+ *HOXA9*-3′-UTR -mutant	3	1080.374±121.249
lipo2000+ *HOXA9*-3′-UTR	3	1197.352±162.872
lipo2000+*HOXA9*-3′-UTR-mutant	3	1107.123±30.490
F	168.622	(welch)
P	<0.001	

* vs. miR-196b mimics control+*BCR-ABL1*-3′-UTR, P = 0.006; * vs. miR-196b mimics+ *BCR-ABL1*-3′-UTR -mutant, P<0.001; * vs. lipo2000+ *BCR-ABL1*-3′-UTR, P<0.001; * vs. lipo2000+*BCR-ABL1*-3′-UTR -mutant, P = 0.001 (missing case, pairwise comparisons). # vs. miR-196b mimics contro+*HOXA9*-3′-UTR, P<0.001; # vs. miR-196b mimics+ *HOXA9*-3′-UTR -mutant, P = 0.016; # vs. lipo2000+ *HOXA9*-3′-UTR, P = 0.027; # vs. lipo2000+*HOXA9*-3′-UTR -mutant, P<0.001 (missing case, pairwise comparisons).

### Modulation of *BCR-ABL1* and *HOXA9* expression by miR-196b small interfering (si)RNA and epigenetic drugs

**Figure 3:**
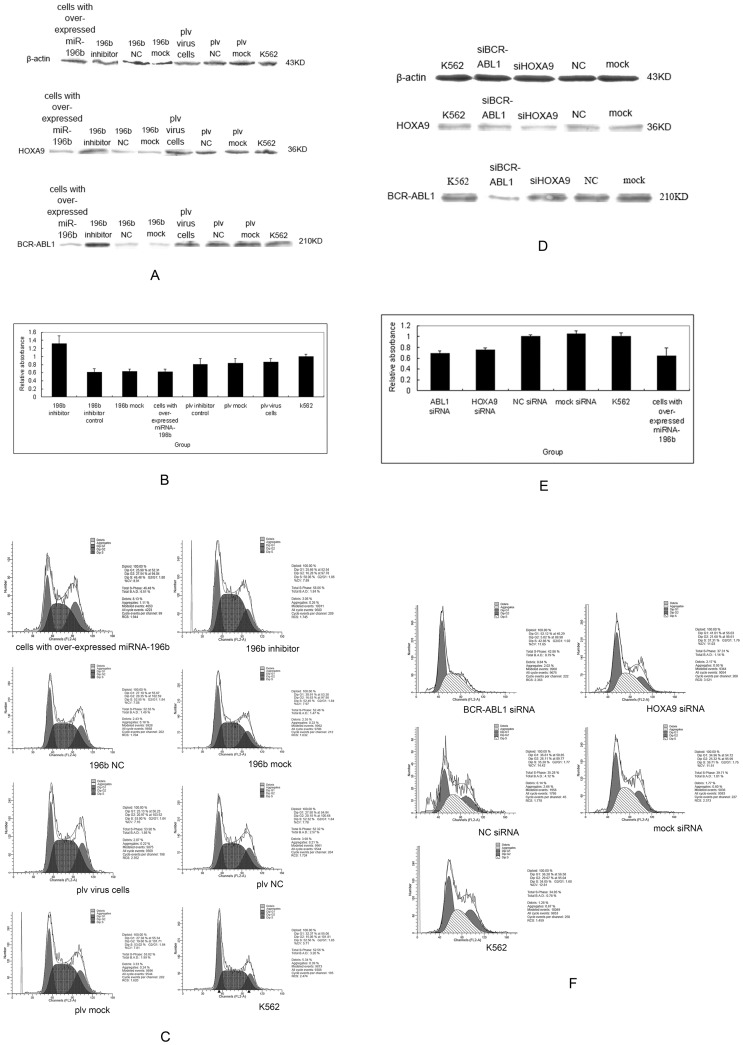
Cell functions after transfection and epigenetic drugs. (A) Protein levels after reintroduction of miR-196b into K562 cells. (B) Proliferation rates of K562 cells after reintroduction or reduction of miR-196b. (C) Cell cycle analysis after reintroduction or reduction of miR-196b. (D) Protein levels after down-regulation of *BCR-ABL1* and *HOXA9* by specific siRNAs. (E) Proliferation rates after down-regulation of *BCR-ABL1* and *HOXA9* by specific siRNAs. (F) Cell cycle analysis after down-regulation of *BCR-ABL1* and *HOXA9* by specific siRNAs. (G) MiR-196b expression in K562 cells after treatment with Aza and PBA separately, or Aza + PBA. (H) BCR-ABL1 and HOXA9 protein levels after treatment with Aza, PBA or Aza + PBA.

Human pre-miR-196b was amplified from normal human bone marrow (Figure 2G). Follow up testing was performed on K562 cells infected by 196b virus (Figure 2H) and pLV virus (Figure 2I). A restoration of the expression of miR-196b reduced BCR-ABL1 and HOXA9 protein levels (Figure 3A). This was accompanied by a dramatic decrease in the cell proliferation rate (Figure 3B, Table 3) and retardation of the G2 stage (Figure 3C). A reduction in the expression of miRNA-196b, in the cells where it had been overexpressed, restored BCR-ABL1 and HOXA9 protein levels (Figure 3A), enhanced cell proliferation (P<0.05) (Figure 3B, Table 3), and retarded the S stage of the cell cycle (Figure 3C). In addition, down-regulation of *BCR-ABL1*, by specific siRNAs, reduced BCR-ABL1 protein levels (Figure 3D) and inhibited proliferation (P<0.05) (Figure 3E, Table 3), as also observed in cells that showed over-expression of miRNA-196b and a retarded G1 stage (Figure 3F). Down-regulation of *HOXA9*, by specific siRNAs, reduced HOXA9 protein levels (Figure 3D) and induced proliferation arrest (P<0.05) (Figure 3E, Table 3), as observed in cells that showed over-expression of miRNA-196b. This also resulted in the retardation of the G1 stage of the cell cycle (Figure 3F).

**Table 3:** Cell proliferation assessed by CCK-8 assay after miR-196b and siRNA transfection.

	n	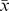 ±*s*
miR-196b inhibitor	3	3.674±0.080*
miR-196b inhibitorcontrol	3	1.690±0.035
miR-196b mock	3	1.703±0.018
cells with over-expressedmiRNA-196b	3	1.716±0.079
K562	3	2.611±0.103
F	336.464	(welch)
P	<0.001	
*BCR-ABL1* siRNA	3	1.821±0.063**
*HOXA9* siRNA	3	1.939±0.079^#^
NC siRNA	3	2.717±0.126
mock siRNA	3	2.654±0.075
K562	3	2.675±0.129
cells with over-expressedmiRNA-196b	3	1.676±0.060
F	69.425	(welch)
P	<0.001	

* vs. miR-196b inhibitor control, P = 0.001; * vs. miR-196b mock, P = 0.003; * vs. cells with over-expressed miRNA-196b, P<0.001; * vs. K562, P = 0.002; cells with over-expressed miRNA-196b vs. K562, P = 0.004 (missing case, pairwise comparisons). ** vs. *HOXA9* siRNA, P = 0.851; ** vs. K562, P = 0.032; **vs. cells with over-expressed miRNA-196b, P = 0.495; # vs. K562, P = 0.034; # vs. cells with over-expressed miRNA-196b, P = 0.166 (missing case, pairwise comparisons).

Promoter methylation can be reversed by specific epigenetic drugs. We treated K562 cells with Aza, PBA and Aza + PBA, which resulted in partial but efficient demethylation of the miR-196b promoter. The miR-196b expression was restored in K562 cells that were treated with Aza + PBA but not in those treated with Aza and PBA, separately (Figure 3G). The three treatment groups showed a significant reduction in both BCR-ABL1 and HOXA9 protein levels (Figure 3H).

## Discussion

The levels of expression of miR-196b were significantly lower in CML patients than in healthy controls. The methylation of the miR-196b promoter CpG islands was also increased in CML patients, which implied that expression levels and promoter methylation status could be linked. The differences observed between CML patients and healthy individuals correlate to the mechanisms of action of many tumor suppressor genes [21], [22], which indicates that miR-196b could be involved in the mechanism that underlies the development of CML. The regulation of miRNA-196b by DNA methylation has been shown to be involved in the development of many other cancers and may be significant in CML. For example, Tsai KW et al. demonstrated that abnormal DNA hypomethylation induces the overexpression of miR-196b in gastric cancer [23]. Hulf T et al. found that miR-196b was repressed by epigenetic activity in prostate cancer cells [24]. Abe W et al. suggested that the expression of miR-196b in endometriotic cyst stromal cells is repressed by hypermethylation of the DNA of the miR-196b gene [25]. Schotte D et al. demonstrated that up-regulation of miR-196b coincides with reduced DNA methylation at CpG islands in the promoter regions of miR-196b in pediatric ALL [26]. The differences in levels of miR-196b and methylation that were observed between ALL and CML require further studies to identify whether the discrepancy was related to different sub-types of cancer, different stages of cancer or differences between adults and children.

Statistical analyses also indicated that, unlike CML, there were no significant differences in the methylation status of the miR-196b promoter CpG islands between healthy controls and patients with other types of leukemia. The differences between types of leukemia were also not significant. Further studies are needed to determine if this difference is related to the fact that CML involves the increased proliferation of completely differentiated cells, and results from abnormal signal transduction or incontrollable cell proliferation [27], rather than representing a cell-maturity disorder, as in ALL.

The BCR-ABL1 and HOXA9 protein levels were reduced in K562 cells that were treated with demethylating agents. These results indicated that a reduction in the methylation of the miR-196b promoter could inhibit BCR-ABL1 and HOXA9 protein expression. The use of demethylating agents, including Aza and PBA, could provide a potential treatment for CML. However, miR-196b expression was not restored in cells treated with Aza and PBA, separately, whilst it was restored in cells treated with a combination of Aza + PBA. This may indicate that the mechanism of change of miR-196b expression involved not only hypermethylation, but also excessive deacetylation [28].

The luciferase assay confirmed that *BCR-ABL1* and *HOXA9* are target genes of miR-196b. Western blotting indicated that the reintroduction of miR-196b, into K562 cells, reduced BCR-ABL1 and HOXA9 protein levels. The reduction of miRNA-196b expression restored BCR-ABL1 and HOXA9 protein levels. These data confirmed the roles of *BCR-ABL1* and *HOXA9* as miR-196b target genes. There are no published studies of *BCR-ABL1* as a target for miR-196b, but there are several papers that have shown that HOXA9 is a target gene. For example, Li Z et al. reported that miR-196b directly targets HOXA9/MEIS1 oncogenes in MLL-rearranged leukemia [29]. Shen J et al. showed that the host gene of miR-196b was HOXA9 in hepatocellular carcinoma [30]. Tsai KW et al. reported that miR-196b is located in the HOXA cluster, within exon AB of HOXA9 (chromosome7), in gastric cancer [22].

To date, this is the first report on the effects of miR-196b on cell proliferation in K562 cells. The overexpression of miR-196b was associated with decreased cell proliferation, whilst a reduction of expression enhanced cell proliferation. This indicated that miR-196b is involved in the proliferation of K562 cells. Recent studies have indicated that miR-196b also inhibits cell proliferation and promotes apoptosis in B-cell ALL cells [4], but promotes proliferation and improves survival in gastric cancer cells [23]. Hence, the effects of miR-196b on cell proliferation differ between different cell lines. The *BCR-ABL1* gene is important for regulating cell proliferation in CML [31]. It is translated to produce a fusion protein with abnormal tyrosine kinase activity, which results in hematopoietic stem cell dysregulation, myeloid progenitor cell proliferation and reduced apoptosis. The *HOXA9* gene is necessary for the normal differentiation of hematopoietic cells and *HOXA9* deficiency can result in reduced cell proliferation, differentiation and apoptosis induction [32]. The reintroduction of miR-196b was associated with inhibition of *BCR-ABL1* and *HOXA9* expression and the consequent reduction of their regulatory effects on proliferation. The expression and function of the genes could be restored by reducing the expression of miRNA-196b. In addition, down-regulation of *BCR-ABL1* and *HOXA9,* by specific siRNAs, resulted in the inhibition of cell proliferation, compared with cells that over-expressed miRNA-196b. This demonstrated that the inhibition of proliferation by miR-196b occurred by the inhibition of both *BCR-ABL1* and *HOXA9*.

In conclusion, this study demonstrated that low expression levels of the tumor suppressor, miR-196b, were associated with up-regulation of the oncogenes *BCR-ABL1* and *HOXA9*, ultimately leading to the development of CML. These results verified our previous hypothesis and indicated that miR-196b is a potential target for therapeutic intervention in CML.

## Supporting Information

File S1 **Supporting file containing Tables S1, S2, S3.** Table S1. Primers used for detection of miRNAs and for cloning and mutagenesis of the human *BCR-ABL1* 3′-UTR and *HOXA9* 3′-UTR. Table S2. *BCR-ABL1* and *HOXA9* target sequences for RNA interference. The siRNA ID includes the start nucleotide of the targeted sequence in the reference transcript for the human *BCR-ABL1* gene and *HOXA9* gene. Table S3. Patient samples.
(DOC)
