# Supplementary material for: Low Expression of miR-196b Enhances the Expression of BCR-ABL1 and HOXA9 Oncogenes in Chronic Myeloid Leukemogenesis
Source: PLoS One. 2013 Jul 19;8(7):e68442. doi: 10.1371/journal.pone.0068442 (PMC3716876; doi:10.1371/journal.pone.0068442)
Supplement: File S1 — Supporting file containing Tables S1, S2, S3. Table S1. Primers used for detection of miRNAs and for cloning and mutagenesis of the human BCR-ABL1 3′-UTR and HOXA9 3′-UTR. Table S2. BCR-ABL1 and HOXA9 target sequences for RNA interference. The siRNA ID includes the start nucleotide of the targeted sequence in the reference transcript for the human BCR-ABL1 gene and HOXA9 gene. Table S3. Patient samples. (DOC) [file pone.0068442.s001.doc]

Table S1. Primers used for detection of miRNAs and for cloning and mutagenesis of the human *BCR-ABL1* 3′-UTR and *HOXA9* 3′-UTR

| Locus | Primer ID | Sequence(5’-3’) | Application |
| --- | --- | --- | --- |
| has-miR-196b | has-miR-196b.F | GCAGCACGCTAGGTAGTTTCC | Q-PCR |
|  | has-miR-196b.R | TATCGTTGTTCTCCACTCCTTGAC | Q-PCR |
|  | has-miR-196b.F2 | GAGTTTTAGGGAGGTTTGTAGAGGT | BSP |
|  | has-miR-196b.R2 | TTCCCAAATCTTACAACCCAAC | BSP |
|  | has-miR-196b.F3 | GTTGGGTTGTAAGATTTGGGAA | BSP |
|  | has-miR-196b.R3 | ATCACCRACCAATTCTAATACTACC | BSP |
|  | has-miR-196b.MF | GCGGCGCGTATATTGTTTTTC | MSP |
|  | has-miR-196b.MR | CCCAAACGACCGCAAATACG | MSP |
|  | has-miR-196b.UF | GAAAGTGGTGTGTATATTGTTTTTT | MSP |
|  | has-miR-196b.UR | CCCAAACAACCACAAATACA | MSP |
|  | pre-miR-196b.F | CGACGCGTGCAGGGAAAGCCGAGGTT | Cloning |
|  | pre-miR-196b.R | CCATCGATCGCGATGGCAGGGTTCTC | Cloning |
| has-U6 | has-U6.F | ATTGGAACGATACAGAGAAGATT | Q-PCR |
|  | has-U6.R | GGAACGCTTCACGAATTTG | Q-PCR |
| cel-mir-39 | cel-mir-39.F | GACTTCATCACCGGGTGTAAATC | Q-PCR |
|  | cel-mir-39.R | TATCGTTGTTCTCCACTCCTTGAC | Q-PCR |
| *BCR-ABL1* mRNA 3’UTR | H-*ABL1* 3’UTR.F | CACTCGAGAGTCAGGGGTCAGGTGT | Cloning |
|  | H-*ABL1* 3’UTR.R | ATAGCGGCCGCGAGGACAGATTTGGATTCA | Cloning |
| *BCR-ABL1* mRNA 3’UTR-  Mutant | H-*ABL1* 3’UTR.F2 | GATACCTCGAGAGTCAGGGGTCAGGTGTCAG | Mutagenesis |
|  | H-*ABL1* 3’UTR.R2 | GCAAACGATCCATCAAGAGCTGGACTCCACAGG | Mutagenesis |
|  | H-*ABL1* 3’UTR.F3 | CAGCTCTTGATGGATCGTTTGCACCGCCTGCCCTC | Mutagenesis |
|  | H-*ABL1* 3’UTR.R3 | GTATCGCGGCCGCGAGGACAGATTTGGATTCAAAAAAAACC | Mutagenesis |
| *HOXA9* mRNA 3’UTR | H-*HOXA9* 3’UTR.F | CACTCGAGATTTGGGCTTATTTAGAA | Cloning |
|  | H-*HOXA9* 3’UTR.R | ATAGCGGCCGCCACAACAATTTGGTCAGT | Cloning |
| *HOXA9* mRNA 3’UTR-  Mutant | H-*HOXA9* 3’UTR.F2 | GATACCTCGAGATTTGGGCTTATTTAGAAAAAAGG | Mutagenesis |
|  | H-*HOXA9* 3’UTR.R2 | CATTATAAAATCCATCATTCTTTCCTTTTGTTTTAAGTC | Mutagenesis |
|  | H-*HOXA9* 3’UTR.F3 | GGAAAGAATGATGGATTTTATAATGCACAACTGTTG | Mutagenesis |
|  | H-*HOXA9* 3’UTR.R3 | CATAAACCCATCCATCAAACCTTCTGCACATATGTATAG | Mutagenesis |
|  | H-*HOXA9* 3’UTR.F4 | GTGCAGAAGGTTTGATGGATGGGTTTATGCTTAATTTTAATTG | Mutagenesis |
|  | H-*HOXA9* 3’UTR.R4 | GTATCGCGGCCGCCACAACAATTTGGTCAGTAGGCCTTG | Mutagenesis |

Table S2. *BCR-ABL1* and *HOXA9* target sequences for RNA interference. The siRNA ID includes the start nucleotide of the targeted sequence in the reference transcript for the human *BCR-ABL1* gene and *HOXA9* gene.

| siRNA ID | Gene | Targeted sequence | Reference transcript | Location |
| --- | --- | --- | --- | --- |
| *ABL1*-homo-265 | *BCR-ABL1* | CCGGGTCTTAGGCTATAAT | NM_005157.4；ENST00000318560 | 267-285 |
| *HOXA9*-homo-452 | *HOXA9* | CCGGCCTTATGGCATTAAA | NM_152739.3；ENST00000343483 | 454-472 |

Table S3. Patient samples.

| sample | age | sex | staging | sample | age | sex | staging |
| --- | --- | --- | --- | --- | --- | --- | --- |
| CML1 | 42 | M | primary | ALL1 | 44 | M | primary |
| CML2 | 16 | F | primary | ALL2 | 57 | F | primary |
| CML3 | 16 | M | primary | ALL3 | 38 | F | primary |
| CML4 | 37 | F | primary | ALL4 | 49 | M | primary |
| CML5 | 30 | F | primary | ALL5 | 64 | M | primary |
| CML6 | 81 | M | primary | ALL6 | 52 | F | primary |
| CML7 | 24 | M | primary | ALL7 | 43 | M | primary |
| CML8 | 43 | F | primary | ALL8 | 56 | M | primary |
| CML9 | 24 | F | primary | ALL9 | 47 | M | primary |
| CML10 | 24 | M | primary | ALL10 | 38 | M | primary |
| CML11 | 36 | M | primary | ALL11 | 38 | M | primary |
| CML12 | 36 | M | primary | ALL12 | 30 | M | primary |
| CML13 | 34 | M | primary | ALL13 | 33 | M | primary |
| CML14 | 59 | M | primary | ALL14 | 48 | F | primary |
| CML15 | 53 | M | primary | ALL15 | 47 | F | primary |
| CML16 | 44 | F | primary | Normal1 | 44 | M |  |
| AML1 | 41 | F | primary | Normal2 | 15 | F |  |
| AML2 | 64 | F | primary | Normal3 | 35 | M |  |
| AML3 | 5 | M | primary | Normal4 | 32 | F |  |
| AML4 | 54 | F | primary | Normal5 | 75 | F |  |
| AML5 | 39 | M | primary | Normal6 | 25 | F |  |
| AML6 | 53 | F | primary | Normal7 | 24 | F |  |
| AML7 | 30 | M | primary | Normal8 | 58 | M |  |
| AML8 | 58 | M | primary | Normal9 | 42 | F |  |
| AML9 | 25 | F | primary | Normal10 | 39 | M |  |
| AML10 | 66 | M | primary |  |  |  |  |
| AML11 | 23 | M | primary |  |  |  |  |
| AML12 | 62 | M | primary |  |  |  |  |
| AML13 | 41 | M | primary |  |  |  |  |
| AML14 | 37 | F | primary |  |  |  |  |
